# *In silico* Selection and Experimental Validation of FDA-Approved Drugs as Anti-quorum Sensing Agents

**DOI:** 10.3389/fmicb.2019.02355

**Published:** 2019-10-10

**Authors:** Marta Mellini, Elena Di Muzio, Francesca D’Angelo, Valerio Baldelli, Serena Ferrillo, Paolo Visca, Livia Leoni, Fabio Polticelli, Giordano Rampioni

**Affiliations:** ^1^Department of Science, University Roma Tre, Rome, Italy; ^2^National Institute of Nuclear Physics, Roma Tre Section, Rome, Italy

**Keywords:** *Pseudomonas aeruginosa*, anti-virulence strategy, quorum sensing inhibition, pimozide, *in silico* screening, molecular docking, new therapeutics, PqsR

## Abstract

The emergence of antibiotic resistant bacterial pathogens is increasing at an unprecedented pace, calling for the development of new therapeutic options. Small molecules interfering with virulence processes rather than growth hold promise as an alternative to conventional antibiotics. Anti-virulence agents are expected to decrease bacterial virulence and to pose reduced selective pressure for the emergence of resistance. In the opportunistic pathogen *Pseudomonas aeruginosa* the expression of key virulence traits is controlled by quorum sensing (QS), an intercellular communication process that coordinates gene expression at the population level. Hence, QS inhibitors represent promising anti-virulence agents against *P. aeruginosa*. Virtual screenings allow fast and cost-effective selection of target ligands among vast libraries of molecules, thus accelerating the time and limiting the cost of conventional drug-discovery processes, while the drug-repurposing approach is based on the identification of off-target activity of FDA-approved drugs, likely endowed with low cytotoxicity and favorable pharmacological properties. This study aims at combining the advantages of virtual screening and drug-repurposing approaches to identify new QS inhibitors targeting the *pqs* QS system of *P. aeruginosa*. An *in silico* library of 1,467 FDA-approved drugs has been screened by molecular docking, and 5 hits showing the highest predicted binding affinity for the *pqs* QS receptor PqsR (also known as MvfR) have been selected. *In vitro* experiments have been performed by engineering *ad hoc* biosensor strains, which were used to verify the ability of hit compounds to decrease PqsR activity in *P. aeruginosa*. Phenotypic analyses confirmed the impact of the most promising hit, the antipsychotic drug pimozide, on the expression of *P. aeruginosa* PqsR-controlled virulence traits. Overall, this study highlights the potential of virtual screening campaigns of FDA-approved drugs to rapidly select new inhibitors of important bacterial functions.

Francesca D’Angelo, Institut Pasteur, Paris, France

## Introduction

The long-term use of antibiotics has dramatically accelerated the emergence of multi-drug and even pan-drug resistant bacterial pathogens worldwide, leading to an alarming increase of difficult-to-treat infections. This worrying scenario especially concerns the ESKAPE pathogens (*Enterococcus faecium*, *Staphylococcus aureus*, *Klebsiella pneumoniae*, *Acinetobacter baumannii*, *Pseudomonas aeruginosa*, and *Enterobacter* species), a group of bacteria that “escape” the action of almost all available antibiotics (Rice, 2008; Boucher et al., 2009). The trend toward antibiotic resistance is even more alarming if considering that only a handful of new antibiotics have been approved by the U.S. Food and Drug Administration (FDA) in the last decade, with many companies considering the R&D for new antibiotics a less attractive asset compared to more rewarding therapeutic areas. Indeed, *de novo* antibiotic development requires large investments that might not grant an economic reward due to the short commercial lifespan of antibiotics, caused by the rapid emergence of resistance (Ventola, 2015; Mohr, 2016; Luepke et al., 2017).

The awareness about the risk of antibiotic resistance for human health has increased in parallel with our comprehension of bacterial pathobiology, so that virulence mechanisms are now recognized as molecular targets for the development of novel anti-virulence drugs targeting the infection process rather than bacterial growth (Rasko and Sperandio, 2010). Although resistance mechanisms to anti-virulence drugs have been described (Zhu et al., 1998; Hung et al., 2005; Maeda et al., 2012; Imperi et al., 2019), targeting virulence rather than growth is expected to pose a reduced selective pressure for the emergence of resistance (Allen et al., 2014). In particular, *in vitro* evolution experiments indicate that drug resistant clones are counterselected if “public goods” that are shared among members of a bacterial population are targeted (*e.g.*, toxins, exo-proteases, and siderophores) (Mellbye and Schuster, 2011; Vale et al., 2016). Since drug-resistant strains are likely to emerge only if they gain a “private” advantage over the susceptible population, quorum sensing (QS) is recognized as an ideal target for the development of anti-virulence agents. Indeed, QS is an intercellular communication system based on the production, secretion and reception of signal molecules that coordinate the expression of secreted virulence factors in different bacterial pathogens (Rampioni et al., 2014; Kalia et al., 2019).

The ESKAPE pathogen *P. aeruginosa* is a model organism for the development of anti-virulence drugs targeting QS (Soukarieh et al., 2018b). This Gram-negative bacterium, that is one of the most dreaded nosocomial pathogens and the main cause of death in cystic fibrosis (CF) patients, has recently been included by the World Health Organization in the list of pathogens for which new therapeutic options are urgently needed (Priority 1: Critical)^1^. The ability of *P. aeruginosa* to cause both acute and chronic infections in different districts of the human body mainly relies on its capacity to adapt to the host by fine-tuning the expression of a wide array of virulence factors, many of which are QS-controlled. As a consequence, numerous anti-virulence drugs targeting the *P. aeruginosa* QS circuitry have been identified in recent years, and their ability to reduce *P. aeruginosa* pathogenicity has been confirmed both *in vitro* and *in vivo* (Rampioni et al., 2014; Soukarieh et al., 2018b). Unfortunately, the majority of the QS inhibitors identified to date are not suitable as lead-like compounds for further drug development, mainly due to their cytotoxicity and unfavorable pharmacological properties (Maura et al., 2016; Soukarieh et al., 2018b).

With the aim to identify bioavailable and safe QS inhibitors that can faster move into clinical trials or serve as leads for drug optimization programs, our group recently undertook whole-cell biosensor-based screening campaigns of libraries of FDA-approved drugs. This drug-repurposing approach led to the identification of niclosamide, an anthelmintic drug, and clofoctol, an antibiotic active against Gram-positive bacteria, as potent and safe QS inhibitors targeting the acyl-homoserine lactones (AHL)-based and the 2-alkyl-4(1*H*)quinolone (AQ)-based QS systems of *P. aeruginosa*, respectively (Imperi et al., 2013; D’Angelo et al., 2018). These FDA-approved drugs effectively reduced *P. aeruginosa* pathogenic potential in animal models of infection, hence representing promising candidates for preclinical studies.

In the last decades *in silico* approaches have been proved as valid aids to conventional drug-discovery programs. In particular, virtual screens carried out through molecular docking simulations allow to preselect promising drug candidates in vast libraries of molecules, so that only a reduced number of predicted hits have to be validated by means of *in vitro* experiments. In this way, time and costs associated to conventional screening campaigns are reduced. In addition, docking simulations allow to predict the likely binding mode of candidate hits onto the selected target, providing a molecular basis for their optimization in terms of binding affinity (Reuter et al., 2015).

On this basis, the present study aims at combining the advantages of drug-repurposing and virtual screening approaches to identify FDA-approved drugs targeting the *pqs* QS system of *P. aeruginosa via in silico* molecular docking.

In *P. aeruginosa* the *pqs* QS system is based on the AQs 2-heptyl-4-hydroxyquinoline (HHQ) and 2-heptyl-3-hydroxy-4(1*H*)-quinolone (PQS) as signal molecules. HHQ is synthesized by the enzymes coded by the *pqsABCDE-phnAB* operon, and is converted to PQS by the monooxygenase PqsH. Both HHQ and PQS can bind to and activate the transcriptional regulator PqsR (also known as MvfR), that in the active form binds to the P*pqsA* promoter region and promotes *pqsABCDE-phnAB* transcription. Hence, HHQ and PQS act as autoinducers to accelerate their own synthesis (Bredenbruch et al., 2005; Heeb et al., 2011; Dulcey et al., 2013; Drees and Fetzner, 2015). While the main role of HHQ is to trigger this PqsR-dependent positive feedback loop, the signal molecule PQS and the protein PqsE (the latter coded by the fifth gene of the *pqsABCDE-phnAB* operon) are the main effectors of the *pqs* QS system. Besides activating PqsR, PQS acts as an iron chelator, is required for the biogenesis of outer membrane vesicles, and promotes the expression of virulence genes *via* a PqsR-independent pathway (Bredenbruch et al., 2005; Mashburn and Whiteley, 2005; Diggle et al., 2007; Rampioni et al., 2016; Lin et al., 2017). PqsE is a multifunctional protein that participates in the synthesis of HHQ and positively controls the expression of multiple virulence factors independently of AQs, likely by activating the transcriptional regulator RhlR *via* the production of an uncharacterized signal molecule that links the *pqs* and *rhl* QS systems (Drees and Fetzner, 2015; Hazan et al., 2010; Rampioni et al., 2010; Rampioni et al., 2016; Mukherjee et al., 2018). Overall, the production of PQS and the expression of PqsE require activated PqsR, and consequently PqsR-inhibitors have been shown to attenuate *P. aeruginosa* virulence both *in vitro* and in animal models of infection (Klein et al., 2012; Ilangovan et al., 2013; Zender et al., 2013; Lu et al., 2014; Starkey et al., 2014; Maura and Rahme, 2017; Maura et al., 2017; D’Angelo et al., 2018; Soukarieh et al., 2018a). Since the three-dimensional structure of the PqsR domain that interacts with HHQ and PQS (co-inducer binding domain; CBD) has recently been solved (Ilangovan et al., 2013; Kitao et al., 2018), this QS transcriptional regulator now constitutes an ideal target for the identification of new *P. aeruginosa* anti-virulence drugs *via* molecular docking simulations.

In this study, a virtual screening approach has been used to predict PqsR ligands in a library of 1,467 FDA-approved drugs. The ability of the best 5 hits to decrease P*pqsA* activity and AQs level has been tested in wild type *P. aeruginosa* and in *ad hoc* engineered strains. This process led to the identification of the antipsychotic drug pimozide as a specific PqsR inhibitor. Phenotypic assays showed that pimozide hampers the expression of PqsR-controlled virulence traits, such as the production of the virulence factor pyocyanin, swarming motility and biofilm formation, and docking simulations suggest a possible competition with native AQs for PqsR binding. These results provide a proof-of-concept that the drug-repurposing and virtual screening approaches can be combined to accelerate the selection of anti-QS molecules among FDA-approved drugs.

## Materials and Methods

### Bacterial Strains, Media and Chemicals

Bacterial strains used in this study are reported in Table 1. Bacterial strains were routinely grown at 37°C in Luria-Bertani (LB) broth in shaking conditions, or in LB supplemented with 15 g/L agar.

**TABLE 1:** Bacterial strains used in this study.

***P. aeruginosa* strains**	**Characteristics**	**References**
PAO1	Nottingham collection wild type strain.	
PAO1 pMRP9-1	PAO1 wild type strain carrying a pMRP9 derivative for constitutive expression of GFP; Ap^R^/Cb^R^.	D’Angelo et al., 2018
Δ*pqsR*	PAO1 mutant strain with in frame clear deletion of the *pqsR* gene.	Rampioni et al., 2010
Δ*pqsR* pMRP9-1	PAO1 mutant strain with in frame clear deletion of the *pqsR* gene carrying a pMRP9 derivative for constitutive expression of GFP; Ap^R^/Cb^R^.	D’Angelo et al., 2018
PAO1 P*pqsA*::*lux*	PAO1 wild type strain carrying chromosomal insertion of the P*pqsA*::*lux* transcriptional fusion; Tc^R^.	Fletcher et al., 2007
Δ*pqsA* P*pqsA*::*lux*	PAO1 mutant strain deleted in *pqsA* gene carrying chromosomal insertion of the P*pqsA*::*lux* transcriptional fusion; Tc^R^ (named AQ-Rep).	Diggle et al., 2007
Δ*pqsA* mini-CTX::*lux*	PAO1 mutant strain deleted in *pqsA* gene carrying chromosomal insertion of the mini-CTX::*lux* empty vector; Tc^R^ (named C-Rep).	D’Angelo et al., 2018
Δ*pqsR* (pFD-*pqsABCD*)	PAO1 mutant strain with in frame clear deletion of the *pqsR* gene, carrying the pFD-*pqsABCD* plasmid for PqsR-independent production of AQs; Km^R^.	D’Angelo et al., 2018
Δ*pqsAHR* P*pqsA*::*lux* (pPqsR-6H)	PAO1 triple mutant strain deleted in *pqsA, pqsH* and *pqsR* genes carrying chromosomal insertion of the P*pqsA*::*lux* transcriptional fusion and the pPqsR-6H plasmid for IPTG-inducible expression of PqsR; Tc^R^.	Ilangovan et al., 2013

When required, tetracycline (Tc; 200 μg/mL), isopropyl β-D-1-thiogalactopyranoside (IPTG), dimethyl sulfoxide (DMSO), or synthetic PQS were added to the medium. IPTG, DMSO and synthetic PQS were used at the concentrations indicated in the text. Synthetic PQS stock solution was prepared in MeOH at 20 mM concentration (synthetic PQS was kindly provided by Paul Williams and Miguel Càmara – University of Nottingham, United Kingdom). Ergotamine and pimozide were available in our laboratory as drugs of the PHARMAKON library (10 mM stock solutions in DMSO). Dutasteride, eltrombopag and conivaptan were purchased from Sigma-Aldrich, Carbosynth Ltd., and MCE Medchem Express, respectively, and dissolved in DMSO at 10 mM concentration. Pimozide was also purchased from Sigma-Aldrich for further analyses, and dissolved in DMSO at 40 mM concentration.

### Virtual Screening *via* Molecular Docking

Molecular docking simulations were carried out using DockingApp (Di Muzio et al., 2017), a user friendly interface to the molecular docking program AutoDock Vina (Trott and Olson, 2010), on 1,467 FDA-approved molecules extracted from the DrugBank Database and provided in ready-to-dock format as part of the DockingApp package. DockingApp is a freely available platform-independent application to perform docking simulations and virtual screening using AutoDock Vina. An intuitive graphical user interface facilitates the input phase while an embedded JMol applet allows to visualize and analyse the results. The application comes with the DrugBank set of ready-to-dock FDA-approved drugs for virtual screening and drug-repurposing purposes. In all simulations, the search space (docking grid) included the whole PqsR co-inducer binding domain (CBD) structure, in order to carry out “blind” predictions of the “hit” compounds binding sites. Simulations were first carried out on the apo form of the protein (PDB ID: 4JVC) (Ilangovan et al., 2013), by keeping all protein residues rigid. The ten best-ranking compounds, according to the AutoDock Vina scoring function, were then selected for a refinement round in which molecular docking simulations were carried out allowing flexibility of the residues building up the PqsR binding pocket (*i.e.*, Ile149, Ala168, Val170, Ile186, Leu189, Leu207, Leu208, Phe221, Ile236, Tyr258, Asp264, and Thr265) (Ilangovan et al., 2013). The results of docking simulations were analyzed using the molecular graphics program UCSF-Chimera, version 1.12 (Pettersen et al., 2004).

### Bioluminescence Assay

Analyses of PqsR activity in the presence of potential inhibitors has been performed by using *ad hoc* engineered reporter systems in which bioluminescence emission is proportional to PqsR activity.

The primary screening for potential PqsR inhibitors was performed as previously described (D’Angelo et al., 2018). Briefly, the screening was based on the co-culture of *P. aeruginosa* PAO1 wild type (PAO1) and the reporter strain PAO1 Δ*pqsA* P*pqsA*::*lux* (AQ-Rep). PAO1 and AQ-Rep were grown for 16 h at 37°C with shaking (200 rpm) in LB broth or in LB broth supplemented with 200 μg/L Tc, respectively. After growth, PAO1 and AQ-Rep were washed with sterile saline and mixed into LB broth to a final OD_600_ of 0.03 and 0.1, respectively (wild type/reporter ratio ≈ 1/3). Two-hundred μL aliquots of the diluted co-cultures were dispensed into 96-wells black clear-bottom microtiter plates. All compounds used in the primary screening were dissolved in DMSO to 10 mM concentration. The compounds were added to the microtiter plates containing the co-cultures at the final concentrations of 20 and 200 μM. As untreated controls, the same amount of DMSO alone as in the treated samples was added to the microtiter wells containing the co-culture.

For further analysis with pimozide from Sigma-Aldrich, 100 μL LB-grown aliquots of the PAO1/AQ-Rep co-culture (OD_600_ = 0.06 and 0.2, respectively) or of other reporter systems indicated in the text (OD_600_ = 0.02) were dispensed into 96-wells black clear-bottom microtiter plates, and 100 μL of pimozide diluted in LB at concentrations ranging from 50 to 400 μM were added to each well. Also in this case, DMSO alone was used as a control.

For all light emission assays, plates were incubated at 37°C with shaking (120 rpm) for 5 h, and then light emission (RLU) and cell density (OD_600_) of the reporter system were recorded by using an automated Spark 10 M luminometer-spectrophotometer (Tecan). Reporter activity was evaluated as Relative Light Units (RLU) normalized to cell density (OD_600_). Alteration in promoter activity induced by the tested compounds was determined by comparing the promoter activity of the specific biosensor system in untreated and treated samples.

### Quantification of AQs

AQ signal molecules in *P. aeruginosa* PAO1 culture supernatants were quantified as previously described (Fletcher et al., 2007). PAO1 wild type cultures were grown at 37°C in 96-well microtiter plates with shaking (120 rpm) in LB broth supplemented with the tested compounds or solvent vehicle (*i.e.*, DMSO) as a control. After 7 h of incubation, cell-free supernatants of PAO1 wild type cultures were collected and 5 μL were added to 195 μL of the AQ-Rep biosensor (OD_600_ = 0.1) dispensed into 96-wells black clear-bottom microtiter plates. Plates were incubated for 5 h at 37°C with shaking (120 rpm), and light emission (RLU) and cell density (OD_600_) of the cultures were recorded by using an automated Spark 10 M luminometer-spectrophotometer (Tecan). A calibration curve was generated by growing the AQ-Rep biosensor strain with synthetic PQS at concentrations ranging from ∼ 45 nM to 300 μM. The resulting dose-response curve was used as a landmark to determine the concentration of the AQs in each culture supernatant.

### Pyocyanin Production, Swarming Motility and Biofilm Formation Assays

The assay for pyocyanin extraction and quantification has been performed as previously described (Essar et al., 1990) on PAO1 wild type and PAO1 Δ*pqsR* strains. Bacterial strains were grown for 16 h at 37°C with shaking (200 rpm) in LB broth in the presence of 100 μM pimozide or 0.25% (v/v) DMSO (solvent vehicle control).

Swarming motility assays were performed on PAO1 wild type and PAO1 Δ*pqsR* by using swarming plates [0.8% (w/v) nutrient broth N.2, 0.5% (w/v) glucose, 0.5% (w/v) bacteriological agar] (Rampioni et al., 2009). Plates were supplemented with 100 μM pimozide or 0.25% (v/v) DMSO (solvent vehicle control). Swarming motility was directly observed at the air-agar interface after 16 h of incubation at 37°C.

The biofilm formation assay was performed in eight-well chamber slides as previously described (Jurcisek et al., 2011; D’Angelo et al., 2018), with minor modifications. Briefly, PAO1 wild type and PAO1 Δ*pqsR* constitutively expressing GFP *via* the pMRP9-1 plasmid (Davies et al., 1998) were inoculated in an eight-well chamber slide at an OD_600_ of 0.02 in 700 μL of M9 minimal medium supplemented with 20 mM glucose as carbon source, in the presence of 0.25% (v/v) DMSO (solvent vehicle control) or 100 μM pimozide. Bacterial cultures were incubated at 30°C for 24 h. Planktonic cells were gently removed and the wells of the chamber slide were rinsed with sterile saline before confocal microscope (Leica TCS SP5) imaging of the bacterial cells adhered to the glass surface.

### Statistical Analysis

For statistical analysis the software GraphPad Prism 5 was used; one-way analysis of variance (ANOVA) followed by Tukey-Kramer multiple comparison tests were performed. Differences having a *p* value < 0.05 were considered statistically significant.

## Results

### Virtual Screening for the Identification of FDA-Approved PqsR Ligands

We performed a virtual screening to select possible PqsR ligands in a library of 1,467 FDA-approved compounds extracted from the DrugBank database^2^ and already provided in ready-to-dock format as part of the DockingApp software package (see section “Materials and Methods,” for details).

PqsR is a multi-domain transcriptional regulator composed by a *N*-terminal helix-turn-helix DNA-binding domain and a *C*-terminal co-inducer binding domain (CBD). Since the three-dimensional structure of full-length PqsR is unavailable, molecular docking simulations were performed based on the crystal structure of PqsR-CBD. The CBD of PqsR has been crystallized in the apo form (PDB ID: 4JVC) or as a complex with the native AQ ligand 2-nonyl-4-hydroxy-quinoline (NHQ) (PDB ID: 4JVD) or with the quinazolinone (QZN) inhibitor 3-NH_2_-7Cl-C9-QZN (PDB ID: 4JVI) (Ilangovan et al., 2013). It has been shown that both the native ligand and the inhibitor bind to the CBD of PqsR in a site consisting of two adjacent pockets: the quinolone ring is accommodated in pocket B, while the aliphatic chain makes hydrophobic interactions with pocket A (Ilangovan et al., 2013). More recently, the benzamide-benzimidazole inhibitor M64 was shown to bind to PqsR-CBD in a similar way as NHQ and 3-NH_2_-7Cl-C9-QZN, with its benzimidazole group bound in pocket B and the phenoxy group occupying pocket A (PDB ID: 6B8A; Kitao et al., 2018). To avoid selection bias, molecular docking simulations were performed on the apo form of PqsR-CBD. In addition, to increase the reliability of the simulations, the docking search space was not restricted to the A and B binding pockets, but extended to the entire PqsR-CBD, *i.e.*, a “blind” docking procedure was carried out. To speed up the process, the initial screening of the 1,467 FDA-approved compounds was carried out by keeping all amino acid residues rigid.

Possible PqsR ligands were ranked based on the predicted binding affinity calculated with the AutoDock Vina scoring function. In the case of multiple ligands with the same binding affinity, these were prioritized based on the size-independent ligand efficiency (SILE) coefficient. SILE is a normalized parameter derived from the ligand efficiency (LE), a predictive measure of the *per*-atom binding affinity of a ligand to its binding partner (Kuntz et al., 1999).

Following the above procedure, the ten best-ranking putative PqsR ligands (predicted binding affinity ranging from -11.2 to -10.0 kcal/mol) were selected for a second round of molecular docking simulations in which residues previously reported to be involved in the binding of the natural ligand NHQ to the PqsR-CBD (Ilangovan et al., 2013) were considered flexible (see section “Materials and Methods,” for details). The five molecules predicted to display the highest affinity to PqsR-CBD with fixed residues ranked in the first five positions also in the analysis with flexible residues, and are listed in Table 2. Conivaptan is an non-peptide inhibitor of the vasopressin receptor subtypes V1a and V2, commonly used to treat euvolemic and hypervolemic hyponatremia (Ferguson-Myrthil, 2010); ergotamine is an alkaloid acting as a serotonin agonist with vasoconstrictor and analgesic properties (Saxena and De Deyn, 1992); eltrombopag, an agonist of the thrombopoietin physiological target, is used for the treatment of thrombocytopenia (McCormack, 2015); pimozide is an antipsychotic drug used to treat schizophrenia, chronic psychosis, Tourette’s syndrome, and resistant tics (Tueth and Cheong, 1993); dutasteride is a 5α-reductase inhibitor used to treat benign prostatic hyperplasia (Azzouni and Mohler, 2012).

**TABLE 2:** Putative FDA-approved ligands of PqsR-CBD identified *via* molecular docking.

**Drug name and property**	**Structure**	**ΔDG^a^**
	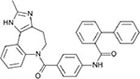	
**Conivaptan:** vasopressin receptors V1a and V2 inhibitor, used for the treatment of euvolemic and hypervolemic hyponatremia		−14.3 (−10.7)
**Ergotamine:** Alkaloid vasoconstrictor used as analgesic		−12.3 (−10.7)
	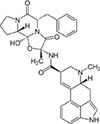	
	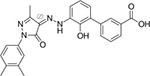	
**Eltrombopag:** Agonist of the Tpo receptor for the treatment of chronic thrombocytopenia		−12.1 (−10.6)
	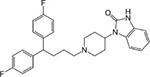	
**Pimozide:** Blocker of dopaminergic receptors used as antipsychotic drug		−12.0 (−10.9)
**Dutasteride:** Oxo-steroid 5-α-reductase inhibitor for the treatment of benign prostatic hyperplasia		−11.3 (−11.2)
	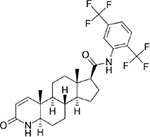	

*^*a*^Δ*G*values (kcal/mol) for drugs binding to the PqsR-CBD apo form (PDB ID: 4JVC; Ilangovan et al., 2013) with flexible residues, predicted by the AutoDock Vina scoring function; the Δ*G* values (kcal/mol) in parenthesis refer to the affinity of the same molecules for the PqsR-CBD obtained in docking simulations with fixed residues.*

**FIGURE 1:**
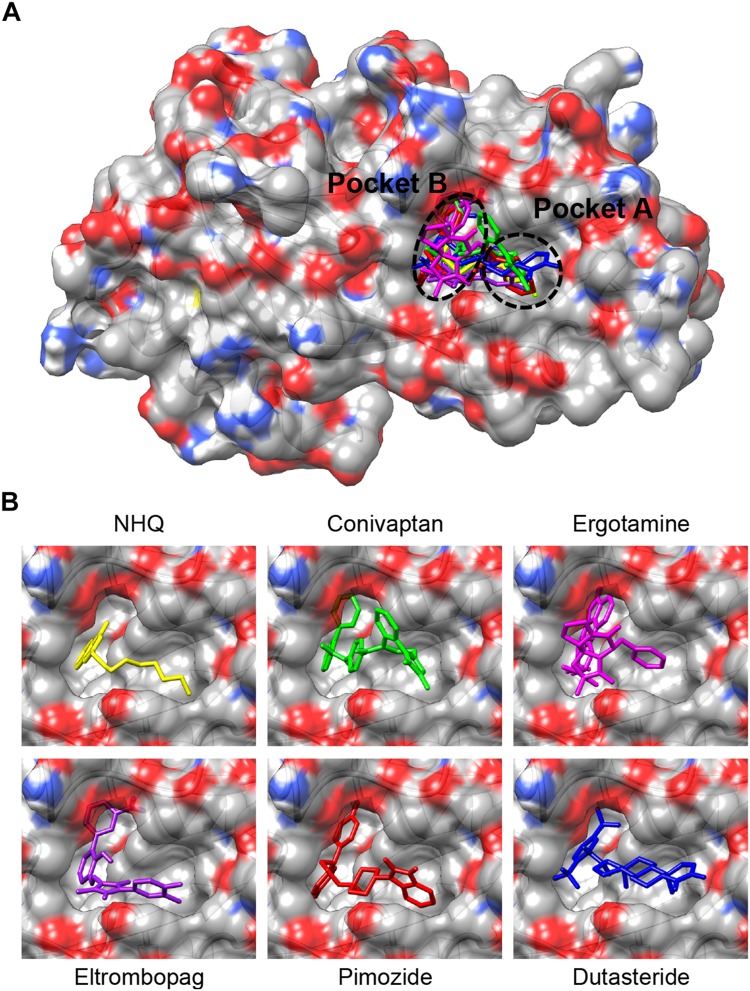
**(A)** Superimposition of the putative complexes formed by conivaptan (green), ergotamine (magenta), eltrombopag (purple), pimozide (red) or dutasteride (blue) with the PqsR-CBD, obtained by molecular docking simulations. Binding of the natural ligand NHQ (yellow) is also shown (Ilangovan et al., 2013). The binding pockets A and B are indicated. **(B)** Enlargement of the ligand-binding site from **(A)**.

Superimposition of the five PqsR-ligand molecular complexes obtained by docking simulations with the crystal structure of the PqsR-NHQ complex (PDB ID: 4JVD) (Ilangovan et al., 2013) predicts that all the five ligands bind in the NHQ binding site, interacting with residues building up the A and B pockets of PqsR-CDB, as observed for the natural ligand NHQ (Figure 1).

### Anti-QS Activity of the Predicted PqsR Ligands

To validate the ability of the predicted PqsR ligands to inhibit PqsR activity in *P. aeruginosa*, the best 5 hits were tested for their ability to reduce bioluminescence in a co-culture system based on wild type *P. aeruginosa* PAO1 (Nottingham collection; herein referred to as PAO1) and isogenic *P. aeruginosa* PAO1 Δ*pqsA* P*pqsA*::*lux* (named AQ-Rep) (Fletcher et al., 2007; D’Angelo et al., 2018). The AQ-Rep biosensor strain is unable to synthesize HHQ and PQS signal molecules as a consequence of *pqsA* mutation, and it carries single-copy chromosomal insertion of a transcriptional fusion between the PqsR-activated promoter P*pqsA* and the *luxCDABE* operon for light emission. Therefore, in the PAO1/AQ-Rep co-culture the AQs produced by PAO1 activate PqsR in AQ-Rep, and consequently promote bioluminescence; hence, a PqsR inhibitor is expected to reduce light emission in this reporter system. The use of the PAO1/AQ-Rep co-culture recently allowed the identification of new PqsR inhibitors (D’Angelo et al., 2018), thus proving its efficacy for the selection of anti-virulence drugs targeting the *pqs* QS system. A co-culture of PAO1 and of a Δ*pqsA* derivative strain carrying the mini-CTX::*lux* empty vector for constitutive light emission (named C-Rep) was used to discriminate between molecules targeting PqsR and molecules affecting light emission in a PqsR-independent way.

The PAO1/AQ-Rep co-culture was incubated with the predicted PqsR-ligands identified *via* the preliminary virtual screening: conivaptan, ergotamine, eltrombopag, pimozide, and dutasteride. Molecules were tested at 20 μM and 200 μM concentrations. Since these drugs were dissolved in DMSO, solvent vehicle control samples in which 0.2% or 2% DMSO alone was added were also analyzed. To ensure specificity, the predicted ligands of PqsR were also analyzed by using the PAO1/C-Rep control co-culture. For each predicted ligand, the residual reporter activity (RLU/OD_600_) was calculated by comparing the reporter activity of the culture grown in the presence of the tested molecule to the reporter activity of the same culture grown in the presence of solvent vehicle, considered as 100%.

As reported in Table 3, 200 μM ergotamine, eltrombopag and pimozide were able to reduce light emission in the PAO1/AQ-Rep co-culture system by 27.9, 30.4, and 46.3%, respectively, while conivaptan and dutasteride did not affect reporter activity. However, 200 μM eltrombopag reduced light emission also in the PAO1/C-Rep control co-culture system (38.5% reduction), suggesting PqsR-independent light inhibition, while ergotamine and pimozide did not significantly alter light emission in the control samples. Notably, ergotamine and pimozide reduced reporter activity at 20 μM concentration (10.1 and 12.3% reduction, respectively), and none of the tested drugs significantly altered *P. aeruginosa* cell density (data not shown). Since ergotamine and pimozide were effective in specifically reducing light emission in the PAO1/AQ-Rep co-culture, without altering bacterial growth, these molecules were selected for further investigations.

**TABLE 3:** Primary and secondary screenings.

**Drug name**	**Residual reporter activity (%)^a^**		**Residual AQ production (%)^b^**	
	**20 μM**	**200 μM**	**20 μM**	**200 μM**
Conivaptan	103.7 (103.1)	100.5 (95.6)	n.d.	n.d.
Ergotamine	89.9 (99.8)	72.1 (98.3)	95.3	93.8
Eltrombopag	99.4 (109.7)	69.6 (61.5)	n.d.	n.d.
Pimozide	87.7 (109.9)	53.7 (99.4)	84.9	50.5
Dutasteride	105.9 (102.4)	111.2 (98.7)	n.d.	n.d.

*^*a*^The first value refers to the residual reporter activity of the PAO1/AQ-Rep reporter system treated with 20 μM or 200 μM of the indicated drug relative to the untreated sample (in this case the same amount of DMSO was used as control). The second value, in brackets, refers to the residual reporter activity of the PAO1/C-Rep control coculture in the same conditions. Activity of the reporter systems in untreated samples are considered as 100%. The average of three independent experiments is reported. ^*b*^Residual production of AQ molecules in *P. aeruginosa* PAO1 cultures treated with 20 μM or 200 μM of the indicated drug relative to the untreated sample (also in this case DMSO was used as control). AQ levels measured in untreated samples are considered as 100%. The average of three independent experiments is reported. n.d., not determined.*

A secondary screening was performed investigating the ability of ergotamine and pimozide to affect the production of the signal molecules AQs. To this end, PAO1 was grown in LB supplemented with ergotamine or pimozide (20 μM and 200 μM) or with DMSO, as a control. The amount of AQs in the corresponding cell-free supernatants was evaluated by using the AQ-Rep biosensor strain, in which light emission is proportional to the amount of AQs present in the medium. As shown in Table 3, ergotamine slightly decreased the level of AQs at both 20 μM and 200 μM (4.7 and 6.2% reduction, respectively), while pimozide reduced the production of these QS signal molecules of 15.1% and 49.5% at 20 μM and 200 μM, respectively.

### Pimozide Inhibits the *pqs* QS System and PqsR-Controlled Virulence Traits

According to the primary and secondary screenings (Table 3) pimozide from the PHARMAKON library reduced P*pqsA* activity in the co-culture system PAO1/AQ-Rep and AQs production in PAO1, without altering bacterial growth. To confirm these data, experiments were replicated with pimozide purchased from a different vendor (Sigma-Aldrich).

**FIGURE 2:**
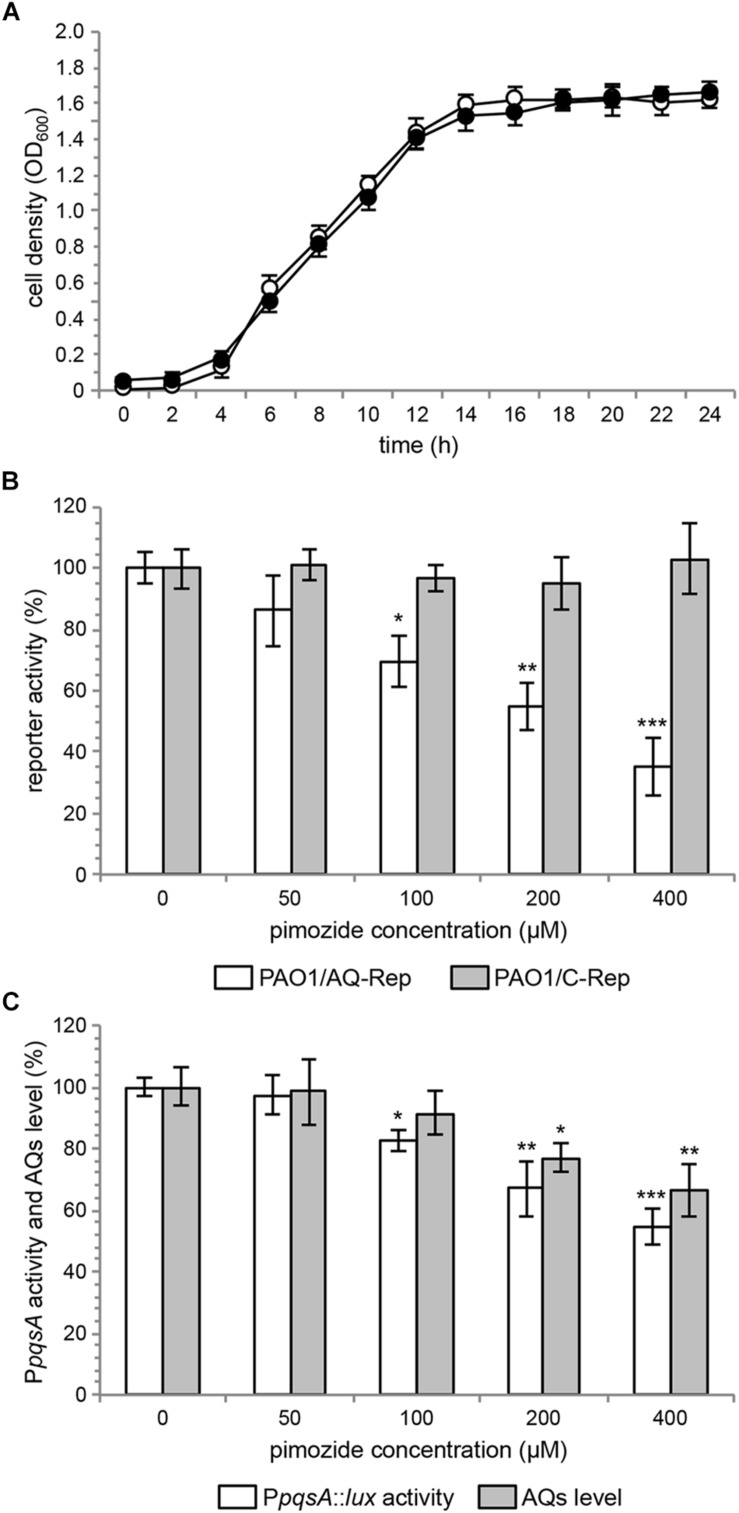
**(A)** Growth curves of PAO1 wild type incubated at 37°C in shaking conditions in LB supplemented with 400 μM pimozide (black circles) or with the corresponding amount of solvent vehicle [*i.e.*, 1% (v/v) DMSO] (open circles). **(B)** Activity of the PAO1/AQ-Rep (white bars) and PAO1/C-Rep (gray bars) co-cultures treated with the indicated concentrations of pimozide. Bioluminescence of the untreated co-cultures normalized to cell density are considered as 100%. **(C)** P*pqsA*::*lux* activity (white bars) and AQ production (gray bars) in PAO1 treated with the indicated concentrations of pimozide. P*pqsA* activity and AQ level measured in untreated PAO1 normalized to cell density are considered as 100%. For **(A–C)**, the average of three independent experiments is reported with *SD*. ^∗^*p* < 0.05; ^∗∗^*p* < 0.01; ^∗∗∗^*p* < 0.001 (ANOVA).

Growth curves reported in Figure 2A show that up to 400 μM pimozide does not alter the growth profile of PAO1. Data reported in Figure 2B confirmed that pimozide significantly reduces light emission in the PAO1/AQ-Rep co-culture in a dose-dependent manner. In detail, 100 μM, 200 μM and 400 μM pimozide significantly reduced PAO1/AQ-Rep activity of 30.1, 45.2, and 64.7%, respectively (Figure 2B, *white bars*). Hence, the decrease in light emission from the co-culture system was comparable to what previously observed for 200 μM pimozide in the primary screening (reduction of PAO1/AQ-Rep activity = 46.3%; Table 3). Conversely, pimozide did not affect bioluminescence in the PAO1/C-Rep control system up to 400 μM (Figure 2B, *gray bars*).

Pimozide was effective in reducing the activity of the PqsR-controlled P*pqsA* promoter in a dose-dependent manner also in PAO1 wild type, with reductions in bioluminescence emission of 17.6%, 33.1 and 45.5% for pimozide concentrations of 100 μM, 200 μM, and 400 μM, respectively (Figure 2C, *white bars*).

To validate the results of the secondary screening, PAO1 was grown in the absence or in the presence of pimozide at different concentrations, and AQ levels were measured in cell-free supernatants by means of the AQ-Rep biosensor strain. In these conditions, pimozide significantly decreased AQ production in a dose-dependent manner only at 200 μM (22.9% reduction) and 400 μM (33.8% reduction) (Figure 2C, *gray bars*).

**FIGURE 3:**
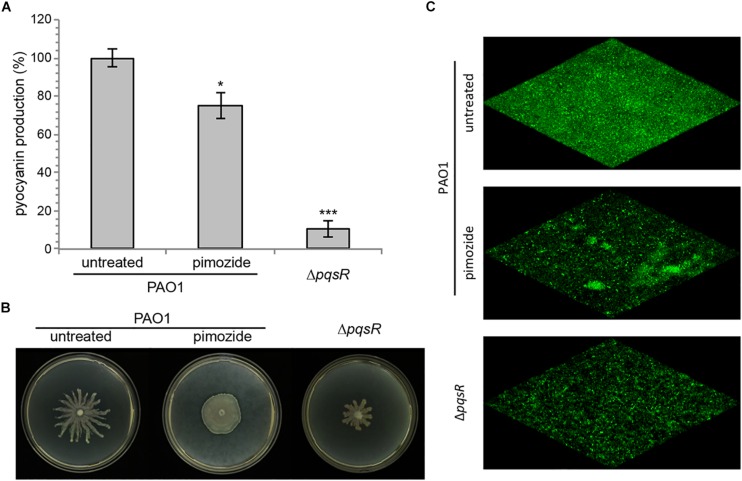
Effect of 100 μM pimozide on pyocyanin production **(A)**, swarming motility **(B)**, and biofilm formation **(C)** in PAO1. The same phenotypes were evaluated in the Δ*pqsR* mutant as a control. For pyocyanin production, the average of three independent experiments is reported with *SD*. ^∗^*p* < 0.05; ^∗∗∗^*p* < 0.001 (ANOVA). For swarming motility and biofilm formation, representative pictures of three independent experiments are shown.

Due to its ability to hamper the *pqs* QS signaling system, pimozide is expected to reduce the expression of *pqs*-controlled virulence traits, such as pyocyanin production, swarming motility and biofilm formation. As shown in Figure 3A, 100 μM pimozide reduced pyocyanin production in PAO1 (24.8% reduction of pyocyanin level in pimozide-treated cultures relative to the control sample supplemented with the solvent vehicle DMSO). Moreover, exposure to 100 μM pimozide significantly altered the swarming motility phenotype by abolishing dendrites formation (Figure 3B) and reduced biofilm formation in a PAO1 strain constitutively expressing GFP (Figure 3C). Despite exerting a milder effect, the inhibition exerted by pimozide on the tested phenotypes in wild type PAO1 mimicked *pqsR* deletion (Δ*pqsR* isogenic strain; Figure 3), thus supporting the hypothesis that PqsR is the likely target of pimozide.

### Validation of the Molecular Mechanism of Action of Pimozide

**FIGURE 4:**
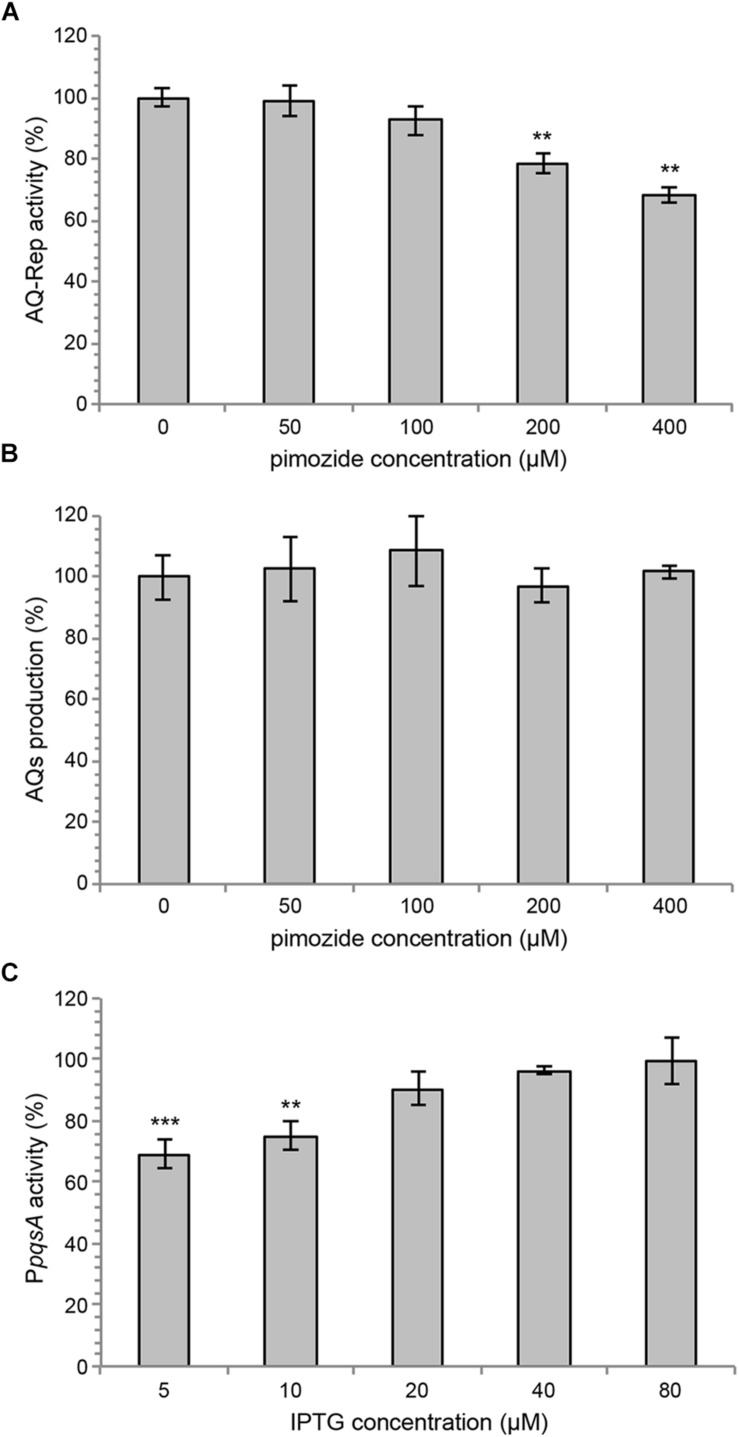
**(A)** P*pqsA* activity in the AQ-Rep biosensor grown at 37°C in shaking conditions in LB supplemented with 5 μM synthetic PQS and the indicated concentrations of pimozide. Biosensor activity in the untreated sample is considered as 100%. **(B)** Production of AQs in *P. aeruginosa* PAO1 Δ*pqsR* (pFD-*pqsABCD*) grown for 16 h in LB in the absence or in the presence of pimozide. The AQ level measured in the untreated sample is considered as 100%. **(C)** Effect of 100 μM pimozide on P*pqsA*::*lux* activity in the PAO1 Δ*pqsAHR* triple mutant carrying the pPqsR-6H plasmid, grown in LB supplemented with 5 μM PQS and different concentrations of IPTG, as indicated in the graph. For **(A–C)**, the average of three independent experiments is reported with *SD*. ^∗∗^*p* < 0.01; ^∗∗∗^*p* < 0.001 (ANOVA).

The inhibitory activity exerted by pimozide on P*pqsA* activity, combined with the reduction of AQs level and attenuation of *pqs-*dependent virulence traits, does not allow to rule out the possibility that pimozide affects AQs biosynthesis instead of, or in addition to, AQs reception by PqsR. To tackle this issue, the effect of pimozide on P*pqsA* promoter activity has been tested in the AQ-Rep biosensor strain grown in the presence of 5 μM synthetic PQS. As shown in Figure 4A, pimozide reduced P*pqsA* activity also in this experimental setting, in which the AQ molecule PQS required to activate PqsR is not endogenously produced by PAO1. Secondly, AQs production was measured in a PAO1 Δ*pqsR* mutant strain carrying the pFD-*pqsABCD* plasmid for constitutive expression of the AQs biosynthetic enzymes. In this genetic background, in which AQ synthesis is PqsR-independent, pimozide did not reduce AQ levels (Figure 4B), indicating that this drug does not affect the activity of the AQs biosynthetic enzymes. Taken together, these experiments indicate that pimozide targets the PqsR-dependent AQs response rather than AQs biosynthesis.

To further support target specificity, the effect of pimozide on P*pqsA* activity was evaluated in a *P. aeruginosa* recombinant strain with tunable levels of PqsR, named PAO1 Δ*pqsAHR* P*pqsA*::*lux* (pPqsR-6H). This strain carries the P*pqsA*::*lux* transcriptional fusion and deletion of the *pqsA*, *pqsH* and *pqsR* genes, therefore it does not synthesize AQs and does not produce the native PqsR regulator, which can be expressed upon IPTG induction *via* the pPqsR-6H plasmid. Therefore, in the absence of IPTG and in the presence of synthetic PQS, the PAO1 Δ*pqsAHR* P*pqsA*::*lux* (pPqsR-6H) strain should express basal level of active PqsR, and the effect of a PqsR inhibitor on P*pqsA* activity should be maximal due to target paucity. Conversely, increasing concentrations of IPTG in the presence of synthetic PQS should result in increased levels of active PqsR, thus reducing the effect of PqsR inhibitors due to increased target abundance. As shown in Figure 4C, in this recombinant strain the repressive effect exerted by 100 μM pimozide on P*pqsA* activity was apparent only for IPTG concentrations ≤ 10 μM, while pimozide had no significant effect on the P*pqsA* promoter for IPTG concentrations ≥ 20 μM. These observations support the hypothesis that pimozide is a ligand and an inhibitor of PqsR.

**FIGURE 5:**
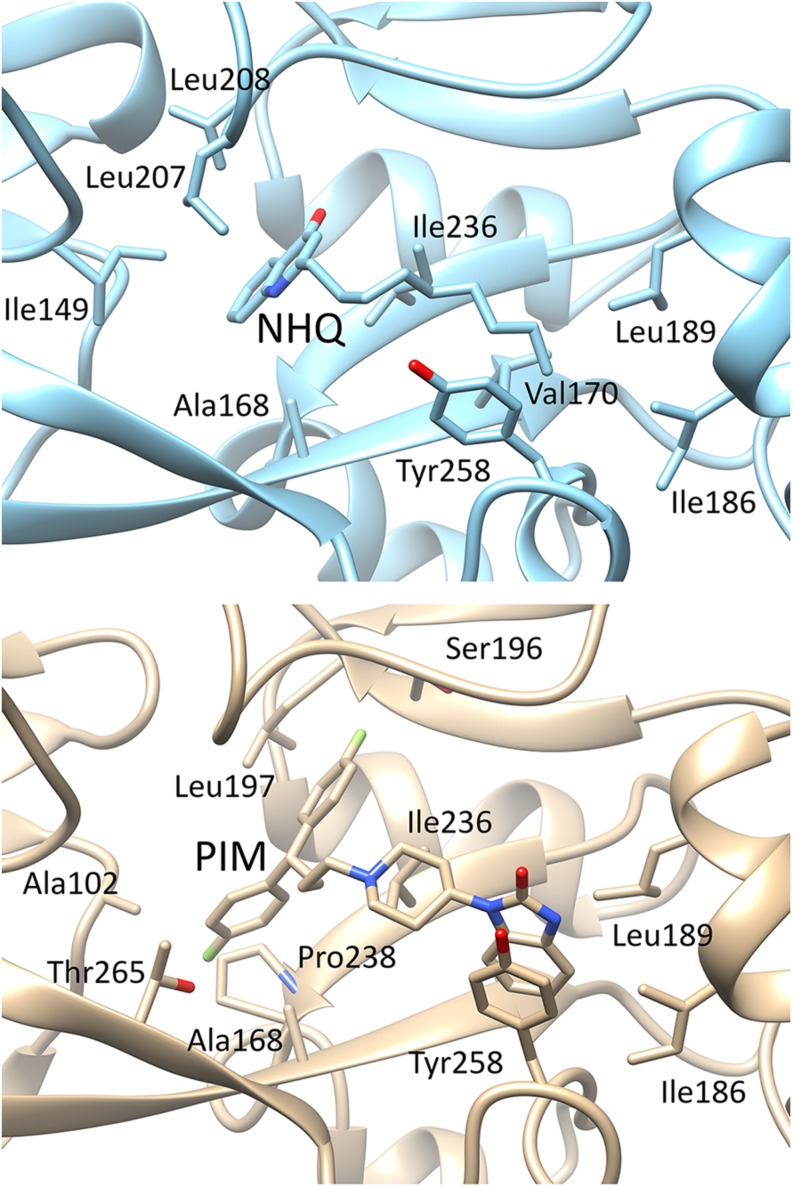
Details of the possible interactions of NHQ (**top panel**; PDB code 4JVD) and pimozide (**bottom panel**; predicted) with the PqsR-CBD binding site. The orientation of the macromolecule is the same as in Figure 1; pocket B is on the left side of the macromolecule and pocket A on the right side.

As shown in Figure 5, pimozide is predicted to bind to the PqsR CBD establishing mainly hydrophobic interactions that closely match those of the natural ligand NHQ (Ilangovan et al., 2013), at least as far as pocket A is concerned. Slightly different interactions are instead observed in pocket B, likely due to a rearrangement of the pocket residues needed to accommodate the bulkier bis(fluorophenyl) moiety of pimozide. Interestingly, pimozide binding to pocket A is predicted to be stabilized also by a π-stacking interaction between the drug benzimidazole group and the Tyr258 aromatic ring (Figure 5, bottom panel), an interaction that mimics the π-stacking interaction experimentally observed between the phenoxy group of the PqsR competitive inhibitor M64 and Tyr258 (Kitao et al., 2018).

## Discussion

Anti-virulence drugs that do not affect bacterial growth hold promise as new therapeutic agents since they are expected to decrease bacterial adaptability to the host environment and to pose a reduced selective pressure for the emergence of resistance with respect to antibiotics. Moreover, virulence mechanisms are often pathogen-specific, thus anti-virulence drugs could avoid dysbiosis usually associated to antibiotic treatments (Rampioni et al., 2014, 2017; Monserrat-Martinez et al., 2019).

The *pqs* QS system controls the expression of multiple virulence factors and biofilm formation, so that *P. aeruginosa* mutants defective in the *pqs* QS system display attenuated pathogenicity in different plant and animal models of infection (Cao et al., 2001; Déziel et al., 2005; Xiao et al., 2006; Lesic et al., 2007; Rampioni et al., 2010; Dubern et al., 2015). Notably, the *pqs* QS system is active during the infection (Collier et al., 2002; Barr et al., 2015), and while *P. aeruginosa* mutants impaired in the *las* QS system are frequently isolated from CF patients (Hoffman et al., 2009; Feltner et al., 2016), the highest proportion of *P. aeruginosa* strains isolated from CF lung are proficient for AQs production (Guina et al., 2003; Jiricny et al., 2014). Moreover, AQ-based QS systems have not been described in the human microbiota so far, suggesting that drugs targeting PqsR should exert a limited effect on the host microbiota. Intriguingly, recent reports indicate that the *pqs* QS system might contribute to the RhlR-dependent activation of virulence genes in the absence of functional LasR (Chen et al., 2019; Kostylev et al., 2019), and that this compensatory role might involve a yet uncharacterized signal molecule produced by PqsE and perceived by RhlR in addition to C4-HSL (Mukherjee et al., 2018). Therefore, by hampering PqsE expression, PqsR inhibitors would impact on virulence factors controlled by both the *pqs* and the *rhl* QS systems, and could be particularly active against *las*-deficient strains emerging during chronic infection in CF patients. On this basis, many inhibitors of the *pqs* QS system have been described in the last decade, proving the ability of anti-*pqs* drugs to reduce the expression of *P. aeruginosa* virulence traits both *in vitro* and in animal models of infection (Calfee et al., 2001; Lesic et al., 2007; Klein et al., 2012; Storz et al., 2012; Ilangovan et al., 2013; Sahner et al., 2013; Weidel et al., 2013; Zender et al., 2013; Lu et al., 2014; Starkey et al., 2014; Sahner et al., 2015; Ji et al., 2016; Thomann et al., 2016; Maura and Rahme, 2017; Maura et al., 2017; D’Angelo et al., 2018; Soukarieh et al., 2018a).

Despite the promise of anti-*pqs* agents for the treatment of *P. aeruginosa* infection, none of these molecules has entered clinical trials so far, likely due to poor pharmacological properties and to the lack of ADME-TOX studies required for their evaluation in humans (Maura et al., 2016; Soukarieh et al., 2018b). To overcome this limitation, we recently exploited a drug-repurposing strategy for the identification of anti-*pqs* drugs *via* whole-cell biosensor-based screening. This strategy succeeded in identifying the FDA-approved drugs clofoctol, miconazole and clotrimazole as new inhibitors of PqsR (D’Angelo et al., 2018).

Most *pqs*-inhibitors have been identified *via* costly and time-consuming biosensor-based screenings or *via* the rational design and experimental validation of AQ analogs or precursors based on the structure of PqsR and of AQ biosynthetic enzymes. Virtual screenings could reduce the time and costs associated to conventional drug discovery programs, hence *in silico* techniques have been extensively applied for the identification of molecules hampering the *las* QS system of *P. aeruginosa* (Yang et al., 2009; Skovstrup et al., 2013; Tan et al., 2013; Soheili et al., 2015; Gökalsın et al., 2017; Kalia et al., 2017; Xu et al., 2017) or QS systems in other bacteria (Zhu et al., 2012; Ali et al., 2018; Ding et al., 2018, 2019; Medarametla et al., 2018). To the best of our knowledge, only synthetic quinoline-based molecules have so far been identified as PqsR antagonists by means of *in silico* docking analyses (Soukarieh et al., 2018a).

In this study we combined the advantages of drug-repurposing and *in silico* screening approaches by exploiting recent knowledge of PqsR-CBD structure and availability of advanced molecular docking tools to identify new FDA-approved drugs with anti-*pqs* activity. The virtual screening led to selection of five hits for which high binding affinity for PqsR was predicted, and *in vitro* experiments demonstrated the anti-*pqs* activity of two of them, namely pimozide and ergotamine. Since pimozide showed the highest inhibitory activity, this drug was experimentally characterized. Phenotypic assays showed that exposure of *P. aeruginosa* PAO1 to pimozide decreased key PqsR-controlled virulence determinants, such as AQ signal molecules, pyocyanin, swarming motility and biofilm formation, without altering bacterial growth, as one would expect for an anti-virulence drug. Additional experiments performed with *ad hoc* engineered *P. aeruginosa* strains and refined *in silico* docking simulations suggest that pimozide competes with the natural ligands HHQ and PQS for PqsR binding, hence hampering the activity of the P*pqsA* promoter. Indeed, analysis of the highest ranking pimozide-PqsR docking complex indicated that the drug interacts with the binding pocket occupying the same position of the natural ligand NHQ (Figure 5), and establishes interactions experimentally demonstrated for both the natural ligand and the competitive inhibitor M64 (Ilangovan et al., 2013; Kitao et al., 2018).

The inability of the predicted PqsR ligands conivaptan and dutasteride to hamper the *pqs* QS system in *P. aeruginosa* and to decrease bioluminescence in the control biosensor system may be related to drawbacks typically associated to virtual screening approaches, including cell impermeability to the selected compound or its modification/inactivation by cellular metabolism. This is in line with the notion that hits emerging from *in vitro* screens, as well as from screens employing heterologous organisms, may lack activity or even function as agonists when tested on the target pathogen (Galloway et al., 2011). As an example, the HHQ analog 2-heptyl-6-nitroquinolin-4*(1H)*-one acted as an antagonist in an *Escherichia coli*-based AQ-reporter strain, but as an agonist in *P. aeruginosa* as a consequence of metabolic modification (Lu et al., 2012). However, a subsequent synthetic modification of this molecule resulted in a strong PqsR antagonist also in *P. aeruginosa*, showing that agonists may still prove useful in the search for antagonists (Lu et al., 2014). More often, the inability of selected hits identified *in silico* or *in vitro* to inhibit target functionality in bacterial cell relates to a lack of internalization or to active efflux. This does not seem to be the case for eltrombopag, since the inhibitory effect exerted by this drug on both the specific and control reporter systems indicates its ability to penetrate *P. aeruginosa* cells, suggesting a QS-independent effect on bioluminescence. In a commentary on the use of whole-cell reporter systems for screens of QS inhibitors, the need for adequate control experiments to assess off-target effects of the tested compounds on reporter activity was emphasized (Defoirdt et al., 2013). For example, pyrogallol was reported to act as a potent inhibitor of AI-2 dependent QS in *V. harveyi*, but subsequent experiments revealed that the apparent inhibitory activity of pyrogallol was a side effect of its peroxide-generating activity on the reporter system, rather than on QS itself (Defoirdt et al., 2013). In our case, unspecific effects of eltrombopag on the reporter system may mask its impact on PqsR functionality. Obviously, the possibility that the virtual screening approach could select false positive hits cannot be ruled out. When molecular assays for *in vitro* evaluation of PqsR activity will be available, it will be possible to verify the ability of the five hits identified in this study to hamper PqsR functionality in a cell-free system.

Searching for side activities in FDA-approved drugs represents a shortcut to develop new therapeutic agents, with considerable potential for shortening the time-consuming and expensive hit-to-lead and lead-optimization phases of drug-discovery programs (Rangel-Vega et al., 2015). In the last years an increasing number of studies identified some antibacterial activity in several drugs approved for different purposes, including anticancer, antifungal, cardiovascular and antipsychotic therapies (Miró-Canturri et al., 2019). However, a possible drawback of drug-repurposing approaches relies on the primary activity of the repurposed drug. As an example, the antipsychotic activity of the dopamine antagonist pimozide, clinically used for the treatement of Tourette’s syndrome and schizophrenia (Tueth and Cheong, 1993), could limit its therapeutic use as anti-virulence drug against *P. aeruginosa*. In fact, it has to be considered that, although rarely, pimozide has been associated to potentially serious adverse effects, including arrhythmia, cardiac arrest, seizures, and neutropenia (Singer, 2010). Neutropenia, in particular, is a worrisome adverse effect for patients suffering a bacterial infection. In addition, the peak serum concentration of pimozide in conventional treatment as an antipsychotic drug is in the nanomolar range (Yan et al., 2010), far below the concentration required to inhibit the *pqs* QS system in *P. aeruginosa*. In spite of these limitations, the pimozide molecular scaffold could serve as the basis for chemical modifications aimed at lowering its dopamine antagonistic activity, while improving membrane permeability and affinity for the PqsR active site, in line with the selective optimization of side activity (SOSA) approach (Wermuth, 2006). However, such a hit-to-lead optimization process would partly compromise the advantage of drug reuporisng, since chemical modification of pimozide would invalidate the FDA-approval, with additional pharmacological testing being required by regulatory agencies. It must be recognized that repurposing of old drugs for new therapies can result in seamless adoption into the clinical practice only if their off-target effect overcomes their primary activity.

That said, pimozide has already been repurposed to inhibit *Listeria monocytogenes* virulence by decreasing cell invasion, vacuole escape and cell-to-cell spread in phagocytic host cells (Lieberman and Higgins, 2009), to inhibit the growth of the protozoan parasite *Toxoplasma gondii* (Dittmar et al., 2016) and for the treatment of breast cancer (Dakir et al., 2018; Elmaci and Altinoz, 2018). Notably, pimozide also inhibited Chikungunya virus (CHIKV) replication in a mouse model of Chikungunya infection when administered in combination with the fatty acid synthesis inhibitor 5-tetradecyloxy-2-furoic acid, with low toxicity *in vivo* (Karlas et al., 2016).

In conclusion, despite low potency of pimozide as a *pqs* inhibitor and predictable side-effects due to its primary antipsychotic activity, this study demonstrates for the first time the potential of virtual screening campaigns to rapidly select new FDA-approved QS inhibitors.

## Data Availability Statement

All datasets generated for this study are included in the manuscript/supplementary files.

## Author Contributions

FP and GR conceived the study. FP, GR, LL, and PV designed the experiments and contributed reagents, materials, and analysis tools. MM, ED, FD, VB, and SF performed the experiments. FP, GR, LL, MM, FD, ED, and VB analyzed the data. GR, FP, and MM wrote the manuscript. All authors corrected, amended the draft of the manuscript, and approved the submitted version.

## Conflict of Interest

The authors declare that the research was conducted in the absence of any commercial or financial relationships that could be construed as a potential conflict of interest.

## Footnotes

1 http://www.who.int/en/news-room/detail/27-02-2017-who-publishes-list-of-bacteria-for-which-new-antibiotics-are-urgently-needed

2 https://www.drugbank.ca/
